# Healthy adult vegetarians have better renal function than matched omnivores: a cross-sectional study in China

**DOI:** 10.1186/s12882-020-01918-2

**Published:** 2020-07-11

**Authors:** Kaijie Xu, Xueying Cui, Bian Wang, Qingya Tang, Jianfang Cai, Xiuhua Shen

**Affiliations:** 1 Department of Clinical Nutrition, Xinhua Hospital Affiliated to Shanghai Jiao Tong University, School of Medicine, Shanghai, 200092 China; 2 Department of Nutrition, School of Public Health, Shanghai Jiao Tong University, Shanghai, 200025 China; 3 Department of Nephrology, 4 Clinical Epidemiology Unit, Peking Union Medical College Hospital, and Chinese Academy of Medical Sciences, Beijing, 100730 China

**Keywords:** Vegetarian, Dietary pattern, Renal function, Kidney, Estimated glomerular filtration rate, Urea nitrogen, Serum creatinine, Uric acid

## Abstract

**Background:**

An appropriate diet is an important determinant of kidney health. However, the association between vegetarian diets and renal function is unclear. We aimed to study the association between vegetarian diets and renal function in healthy adults.

**Methods:**

A total of 269 vegetarians and 269 sex- and age-matched nonvegetarian omnivores were enrolled in this cross-sectional study. Basic characteristics and daily dietary intakes were assessed by face-to-face interviews. Blood samples were collected, and renal function was assessed by measuring blood urea nitrogen (BUN), serum creatinine (SCr), uric acid (UA) and the estimated glomerular filtration rate (eGFR). Blood pressure, fasting blood glucose and blood lipid profiles were also assessed.

**Results:**

The average age of the vegetarians was 35.4 ± 8.6 years, 82.2% of whom were female. We evaluated the association between vegetarian diets and renal function using multivariate analysis. Compared with omnivores, vegetarians had lower BUN [β = − 0.63, 95% confidence interval (CI): (− 0.88, − 0.38)], SCr [β = − 2.04, 95% CI:(− 4.10, 0.02)], and UA levels [β = − 15.15, 95% CI: (− 27.81, − 2.50)] and higher eGFRs [β = 4.04, 95% CI: (0.30, 7.78)] after adjusting for sex, age, body mass index (BMI), physical activity, alcohol consumption, smoking status, low-density lipoprotein cholesterol (LDL), high-density lipoprotein cholesterol (HDL), systolic pressure and fasting blood glucose. Further analysis of food composition and renal function showed that dietary fiber intake was significantly negatively associated with BUN [β = − 0.02, 95% CI: (− 0.03, 0.00)], SCr [β = − 0.14, 95% CI: (− 0.25, 0.04)], and UA levels [β = − 0.72, 95% CI: (− 1.36, 0.07)] and positively associated with the eGFR [β = 0.20, 95% CI: (0.00, 0.40)].

**Conclusions:**

Healthy adult vegetarians have better renal function than omnivores, and the higher dietary fiber intake associated with vegetarian diets may contribute to the protective effect on renal function.

## Background

As a common, real-world dietary pattern, the vegetarian diet is an attractive target for study. Previous studies have suggested that vegetarian diets are associated with reduced risks of obesity, cardiovascular disease, metabolic syndrome and some types of cancer due to their higher contents of unsaturated fat, fiber, folic acid, vitamin C, vitamin E and many phytochemicals [1–4]. Moreover, because of the relatively lower intake and unique source of protein, a vegetarian diet may theoretically have some potential effects on renal function [5, 6]. Nevertheless, the association between vegetarian diets and kidney function is controversial due to the limited number of related studies. The most recent cross-sectional study of 55,113 participants revealed a lower prevalence of chronic kidney disease (CKD) among vegetarians than among omnivores [7]. A study of a population in the Middle East and North Africa reported that the lacto-vegetarian dietary pattern might be protective against the occurrence of CKD after 6.1 years of follow-up [8]. Another study in Taiwan showed no difference in renal function between 102 Buddhist nun vegetarians and a matched control group of omnivores [9]. Some prospective studies reported that a vegetarian diet has a protective effect against renal diseases such as kidney stones and kidney cancer and may reduce renal disease mortality, but relationships between vegetarian diets and renal function parameters were not mentioned [10–12].

CKD is a worldwide public health problem associated with a poor prognosis and high mortality [13, 14]. Dietary management plays an important role in the prevention and treatment of CKD [15]. Most previous data regarding diet and kidney health focus on the association between dietary patterns and CKD morbidity. We aimed to fill the gap by focusing on healthy adults without estimated glomerular filtration rate (eGFR) impairment to explore the relationships between a vegetarian dietary pattern and renal function parameters. We analyzed whether a vegetarian diet is associated with renal function despite the influence of blood pressure and glycolipid metabolism. The roles of the duration of vegetarian dietary habits and nutrients in food are also discussed. Our results may provide significant observational evidence for the dietary management of renal function.

## Methods

### Population

**Fig. 1:**
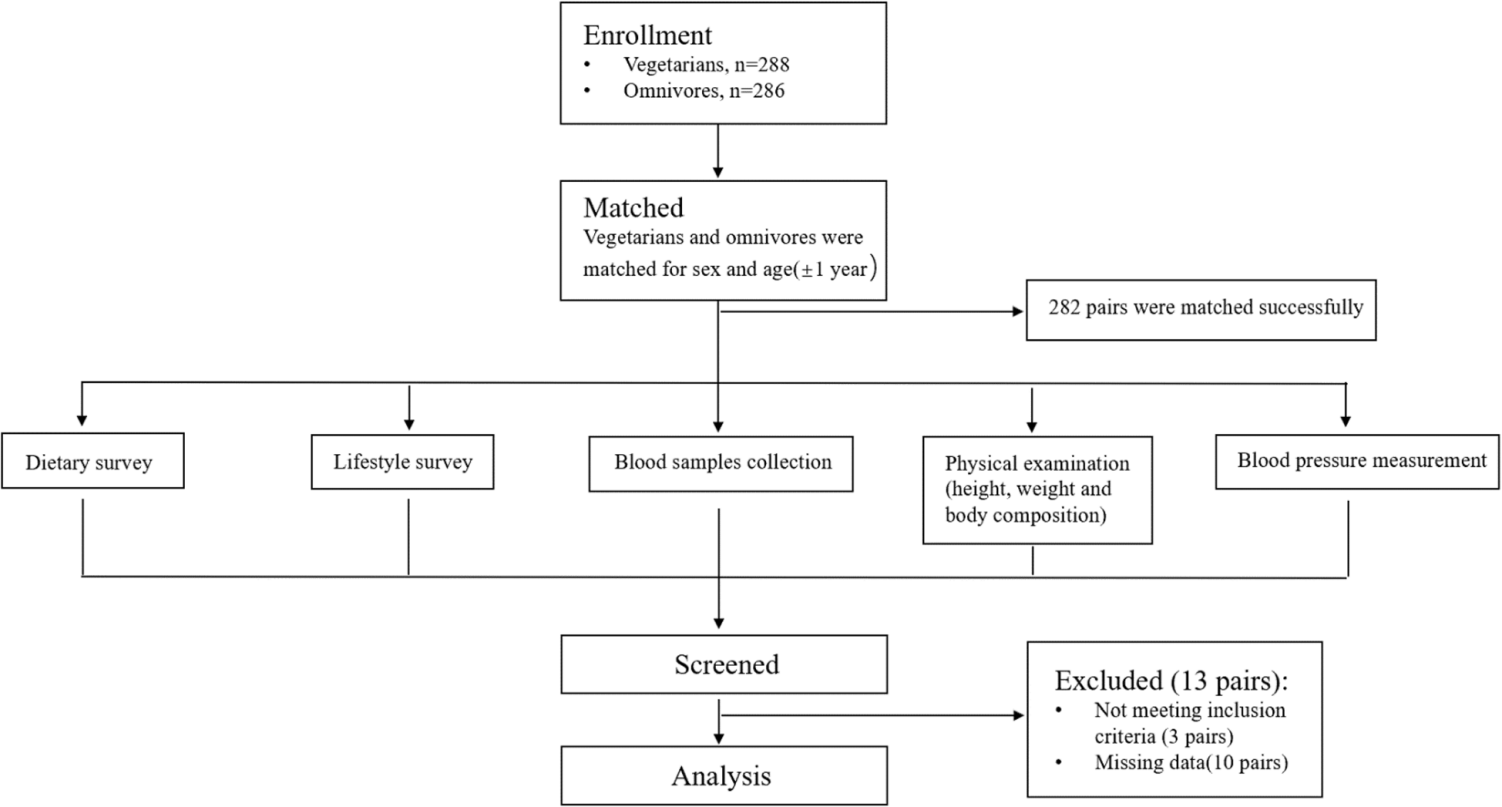
Flowchart of study participants

A total of 538 young (34.5 ± 8.7 years) healthy Chinese adults including 269 vegetarians and 269 sex- and age-matched (± 1 year) omnivores were recruited for this study through online and offline approaches. All subjects were volunteers. The vegetarian subjects were recruited through advertisements with the Vegetarian Society of China, some vegetarian restaurants in Shanghai or other publicity in media, and word of mouth. Once included in our study, the vegetarian subjects were asked to recommended one matched omnivore among her or his friends according to the following criteria: 1) the same sex; 2) the same age or ± 1 year; and 3) a similar lifestyle and social class. The recruitment criteria for participants included 1) adoption of a vegetarian diet for at least 12 months (for vegetarians); 2) residence in Shanghai for more than 6 months; 3) age between 18 and 60 years; 4) the ability to understand the contents of the questionnaires; and 5) no history of pregnancy or breastfeeding within the previous 12 months (for female participants). The exclusion criteria included 1) a diagnosis of any renal disease, acute illness and severe nutritional malabsorption. All the subjects were invited to Xinhua Hospital between March 2015 and May 2016 to participate in this study (see the flowchart in Fig. 1) after providing written informed consent. The study was approved by the Ethics Committee of Shanghai Jiao Tong University, School of Medicine.

### Laboratory tests

After 10–12 h of fasting, venous blood was collected from each participant and then sent to the Clinical Laboratory Center of Shanghai Xinhua Hospital for laboratory tests. Data for fasting blood glucose, blood lipid profiles, including total cholesterol (TC), triglycerides (TGs), low-density lipoprotein cholesterol (LDL), high-density lipoprotein cholesterol (HDL), and the LDL/HDL ratio, and renal function parameters, including blood urea nitrogen (BUN), serum creatinine (SCr), and uric acid (UA), were collected. The eGFR was used to evaluate renal function in terms of excretion and filtration and was calculated based on the Chronic Kidney Disease Epidemiology Collaboration (CKD-EPI) from SCr. Higher eGFR values indicate better renal function, mild renal impairment was defined by an eGFR< 90 mL/minute/1.73 m^2^, and the presence of CKD was defined as an eGFR< 60 mL/minute/1.73 m^2^ [16]. Blood pressure was also measured by an automatic blood pressure machine (OMRON, HEM-759P, Japan).

### Dietary and lifestyle variable assessments

Habitual dietary intakes were assessed in a face-to-face interview using a 24-h recall questionnaire conducted by trained dietitians (The details and of 24 h dietary recall process and questionnaire see the supplement file 1adn 2). We used the 24-h dietary recall questionnaire from “Dietary survey method part of Sanitary Industry Standards of the People’s Republic of China(WST426.1—2013)”, which was widely used in China, including “Chinese National Nutrition and Health Survey” [17, 18]. To help subjects recall and estimate their dietary intakes, dietitians provided food images, oral descriptions, and food models as a part of the 24-h dietary recall method. Data entry and calculation of the 24-h dietary recall questionnaire results were performed using Nutrition Calculator v2.5 software developed by the Institute for Nutrition and Food Safety of the Chinese Center for Disease Control and Prevention and Beijing B-win Technology Co., Ltd.

All vegetarians in our study claimed that they had followed a vegetarian diet by consuming no meat, poultry or aquatic products at any meals daily for over a year. Those who did not consume any animal products were defined as “vegans”, while those who consumed eggs and/or dairy products were considered “lacto-ovo vegetarians”. Among the 269 vegetarians, 70 vegans (26.0%) and 199 lacto-ovo vegetarians (74.0%) were noted. Subjects who did not reject consumption of animal products were defined as omnivores.

All participants were required to complete general condition questionnaires via face-to-face interviews. Basic characteristics such as age, sex, income, education level, marital status, tobacco use, alcohol consumption, work intensity, and the frequency, time, and type of physical activity were recorded.

### Physical examination

Height and weight were measured using digital scales to calculate the BMI. We also used a body composition analyzer (Biospace InBody 720, Korea) to detect muscle mass to identify body protein components that could affect renal function indicators, such as creatinine. All the measurements were performed by professional dietitians while the subjects were minimally clothed without shoes.

### Statistical analysis

Data analysis was performed using the Statistical Program for Social Sciences 25.0 (SPSS, IBM, USA). Continuous variables are presented as the means ± standard deviations (SDs) (e.g., age, physical activity, sedentary time, BMI, blood pressure, alcohol consumption, TC, TG, LDL, HDL, the LDL/HDL ratio, BUN, SCr, UA, the eGFR and daily dietary intakes including energy, protein, protein intake/weight, the protein energy supply ratio, calcium, phosphorus, potassium and sodium). Categorical variables (e.g., sex, marital status, regular physical examination, ethnicity, education level, working intensity, income, alcohol use, and mild eGFR impairment) were presented by proportions. To compare differences between the vegetarian group and omnivore group, paired t tests were performed for continuous data, Wilcoxon matched-pairs signed-ranks tests were performed for ordinal variables, and McNemar tests were performed for matched categorical variables. Differences between the vegan group and lacto-ovo vegetarian group were also assessed. We performed two-independent-sample t tests for continuous data and used Wilcoxon rank-sum tests for ordinal variables. χ^2^ tests were performed for categorical variables.

Multivariable-adjusted β coefficients [95% confidence intervals (CIs)] for the associations of vegetarian dietary patterns [omnivore (reference), total vegetarian (lacto-ovo vegetarian and vegan), lacto-ovo vegetarian, and vegan] with renal function parameters (BUN, SCr, UA, and the eGFR) were estimated using linear regression. The covariates were sex, age, BMI, skeletal muscle mass, physical activity, alcohol consumption, smoking status, blood pressure, blood lipid profiles, fasting blood glucose and vegetarian diet duration. The associations between dietary intake compositions and renal function parameters were also estimated using multiple-linear regression. The covariates were sex, age, BMI, skeletal muscle mass, physical activity, alcohol consumption, smoking status, blood pressure, blood lipid profiles, fasting blood glucose and vegetarian diet duration.

All *P* values were calculated based on two-sided tests, and the significance level for each test was set at *P* < 0.05.

## Results

### Basic characteristics of vegetarians and omnivores

The basic characteristics of the study participants are shown in Table 1. In our study, the mean age of the vegetarians was 35.4 ± 8.6 years, and the mean duration of vegetarian dietary habits was 5.4 ± 5.0 years. The proportion of alcohol users was lower in the vegetarian group, and the vegetarian group was characterized by lower consumption of alcohol. Vegetarians spent more time being physically active (1.9 ± 2.5 h/week vs. 1.4 ± 2.0 h/week) and tended to have a lower BMI (20.9 ± 2.6 kg/m^2^ vs. 22.4 ± 3.5 kg/m^2^) and a higher skeletal muscle mass (22.3 ± 4.1 kg vs. 23.3 ± 4.8 kg). Compared with omnivores, vegetarians had a lower systolic blood pressure (108.0 ± 12.7 mmHg vs. 111.6 ± 15.4 mmHg) and lower fasting blood glucose (4.6 ± 0.6 mmol/L vs. 4.8 ± 0.4 mmol/L) as well as better blood lipid profiles, including lower levels of TC and LDL at 4.1 ± 0.8 mmol/L and 2.5 ± 0.6 mmol/L, respectively, and lower LDL/HDL ratios (2.1 ± 0.6 vs. 2.2 ± 0.7).

**Table 1:** Basic characteristics of vegetarians and omnivores

Variables	Vegetarians			
	Vegans	Lacto-ovo vegetarians	Total vegetarians^a^	Omnivores
	*n* = 70	*n* = 199	*n* = 269	*n* = 269
Sex (Females, %)	74.3	85.4	82.2	82.2
Age (years)	37.2 ± 9.2	34.8 ± 8.3	35.4 ± 8.6	34.8 ± 9.4
Vegetarian diet duration (years)	5.4 ± 4.5	5.4 ± 5.2	5.4 ± 5.0	
Income per month (yuan)				
< 3000	17.1	19.2	18.7^b^	26.1
3000 ~ 8000	67.2	68.2	67.9^b^	65.3
> 8000	15.7	12.6	13.4^b^	8.6
Education (%)				
Primary or secondary	21.4	13.1	15.3^b^	17.7
Vocational	18.6	15.2	16.0^b^	17.7
College and above	60	71.7	68.7^b^	64.6
Alcohol consumption (%)				
None or rarely	98.6	93.5^c^	95.1^b^	83.4
Monthly or weekly	0	5.5^c^	4.1^b^	12.4
Daily	1.4	1	0.8^b^	4.2
Smoking (%)	14.3	8	9.7	8.2
BMI (kg/m^2^)	20.5 ± 2.4	21.1 ± 2.7	20.9 ± 2.6^b^	22.4 ± 3.5
Physical activity (hours/week)	2.0 ± 2.9	1.8 ± 2.3	1.9 ± 2.5^b^	1.4 ± 2.0
Skeletal muscle mass (kg)	22.7 ± 4.8	22.2 ± 3.8	22.3 ± 4.1	23.3 ± 4.8
Systolic pressure (mmHg)	108.6 ± 12.3	107.8 ± 12.8	108.0 ± 12.7	111.6 ± 15.4
Diastolic pressure (mmHg)	69.9 ± 9.4	69.8 ± 9.0	69.9 ± 9.1	70.4 ± 11.0
Fasting blood glucose (mmol/L)	4. 6 ± 0.4	4.7 ± 0.7	4.6 ± 0.62	4.8 ± 0.4
TGs (mmol/L)	1.0 ± 0.5	0.9 ± 0.5	1.0 ± 0.5	0.9 ± 0.5
TC (mmol/L)	4.0 ± 0.8	4.1 ± 0.8	4.1 ± 0.8^b^	4.6 ± 0.8
LDL (mmol/L)	2.5 ± 0.6	2.6 ± 0.6	2.5 ± 0.6^b^	2.9 ± 0.7
HDL (mmol/L)	1.3 ± 0.2	1.3 ± 0.3	1.3 ± 0.3	1.4 ± 0.3
LDL/HDL	2.2 ± 0.6	2.1 ± 0.6	2.1 ± 0.6^b^	2.2 ± 0.7

^a^Total vegetarians: lacto-ovo vegetarians and vegans

^b^Statistical significance when comparing vegetarians and omnivores, *P* < 0.05

^c^Statistical significance when comparing vegans and lacto-ovo vegetarians, *P* < 0.05

### Daily dietary intake

Table 2 details the daily dietary intake of nutrients of the participants conforming to each of the different dietary patterns. The 24-h dietary recall results demonstrated a significant difference between vegetarians and omnivores. The dietary structure of vegetarians was characterized by lower energy intake (1501.1 ± 514.2 kcal/d vs. 1757.3 ± 588.9 kcal/d), lower energy supply ratios of protein and fat intake (protein: 12.2 ± 3.2% vs. 15.7 ± 4.3%; fat: 25.48 ± 8.60% vs 33.01 ± 9.93%), and a higher energy supply ratio of carbohydrate intake (60.49 ± 9.66% vs. 49.94 ± 11.85%). In addition, dietary fiber consumption was higher among vegetarians, especially vegans. Regarding mineral substances, vegetarians consumed less dietary calcium, phosphorus and sodium than omnivores (all *P* values < 0.05). Among vegetarians, vegans consumed more dietary energy and protein and had a higher intake of calcium and phosphorus but a lower intake of sodium than lacto-ovo vegetarians (all *P* values < 0.05).

**Table 2:** Daily dietary intakes of vegetarians and omnivores

Variables	Vegetarians			
	Vegans	Lacto-ovo vegetarians	Total vegetarians^a^	Omnivores
	*n =* 70	*n =* 199	*n =* 269	*n =* 269
Energy (kcal/d)	1507.5 ± 555.6	1498.8 ± 500.3^b^	1501.1 ± 514.2^c^	1757.3 ± 588.9
Carbohydrates (g/d)	233.2 ± 103.1	225.2 ± 82.7	227.3 ± 88.3	217.0 ± 78.8
Carbohydrate energy supply ratio (%)	61.6 ± 12.0	60.1 ± 8.7	60.5 ± 9.7^c^	49.9 ± 11.9
Fat (g/d)	38.7 ± 8.7	43.7 ± 21.4^b^	42.4 ± 21.31^c^	65.7 ± 33.0
Fat energy supply ratio (%)	23.3 ± 9.5	26.2 ± 8.2^b^	25.48 ± 8.60^c^	33.0 ± 9.9
Protein (g/d)	48.7 ± 22.1	45.1 ± 19.1^b^	46.0 ± 19.9^c^	70.5 ± 33.9
Protein energy supply ratio (%)	12.9 ± 3.8	12.0 ± 2.9^b^	12.2 ± 3.2^c^	15.7 ± 4.3
Protein intake/weight (g/kg)	0.9 ± 0.4	0.8 ± 0.4^b^	0.8 ± 0.4^c^	1.2 ± 0.5
Dietary fiber (g/d)	17.29 ± 9.11	14.63 ± 9.3^b^	15.3 ± 9.3^c^	11.83 ± 6.90
Calcium (mg/d)	496.2 ± 316.3	441.6 ± 250.4^b^	455.8 ± 269.5^c^	539.5 ± 340.1
Phosphorus (mg/d)	841.5 ± 367.0	768.4 ± 331.2^b^	787.4 ± 341.7^c^	989.0 ± 378.6
Potassium (mg/d)	2118.1 ± 996.1	1741.3 ± 762.3	1839.4 ± 844.0	1943.3 ± 826.5
Sodium (mg/d)	2389.1 ± 1166.2	2780.0 ± 1263.4^b^	2678.3 ± 1248.6^c^	3767.6 ± 1584.7

^a^Total vegetarians: lacto-ovo vegetarians and vegans

^b^Statistical significance when comparing vegans and lacto-ovo vegetarians, *P <* 0.05

^c^Statistical significance when comparing vegetarians and omnivores, *P <* 0.05

### Renal function parameters

**Fig. 2:**
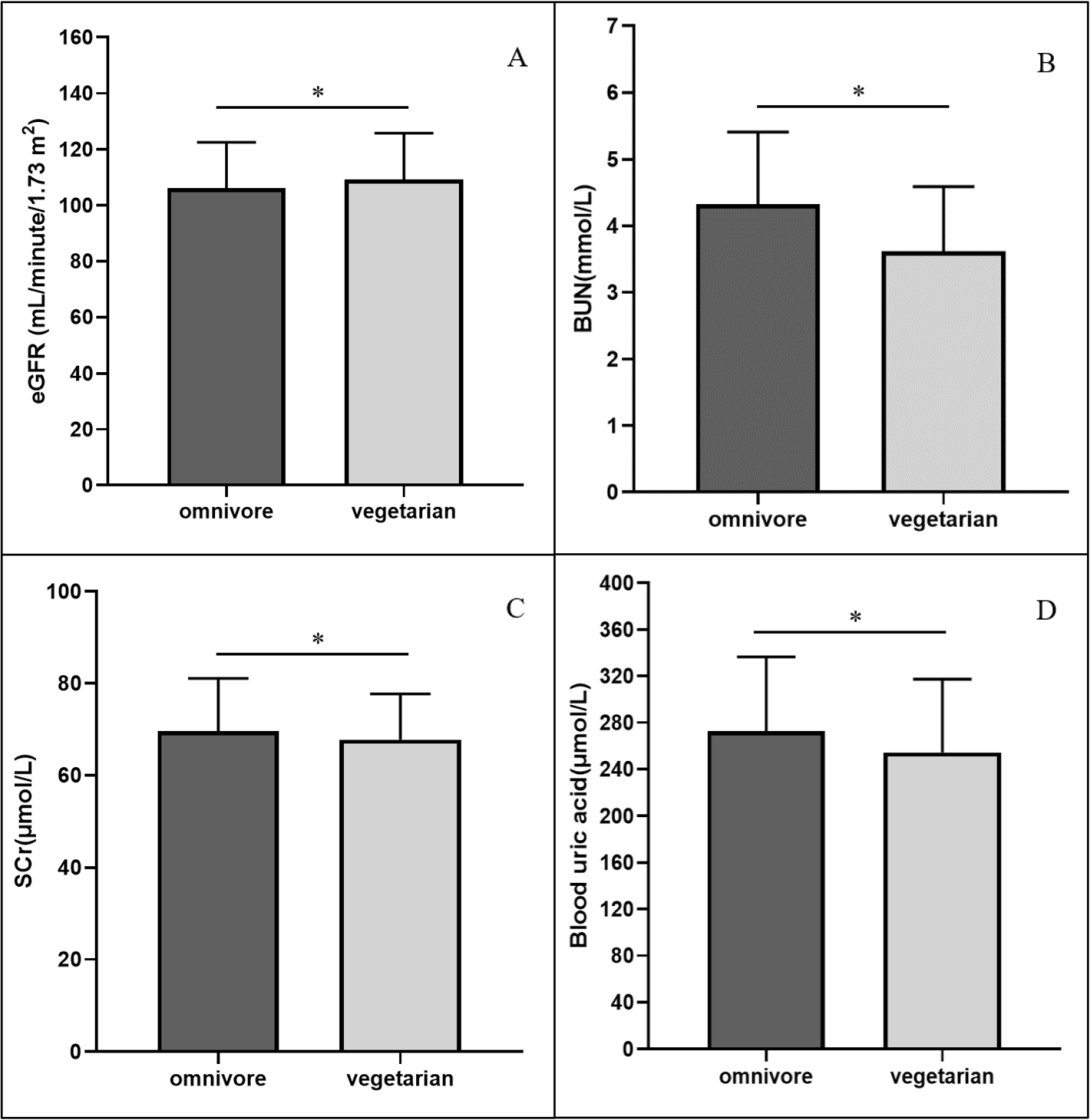
Renal function parameters of omnivores and vegetarians **a**: eGFR of omnivores and vegetarians; **b**: BUN of omnivores and vegetarians; **c**: SCr of omnivores and vegetarians; **d**: UA of omnivores and vegetarians; values are means ± SD. * Statistical significance when comparing vegetarians and omnivores, *P* < 0.05

Figure 2 demonstrates the renal function parameters of omnivores and vegetarians. No subjects had been diagnosed with CKD, and the proportion of mild eGFR impairment was not different between vegetarians and omnivores (13.8% vs. 11.2%) or within vegetarians. The eGFR was higher in vegetarians (109.2 ± 16.6 mL/minute/1.73 m^2^) than in omnivores (106.2 ± 16.4 mL/minute/1.73 m^2^). Vegetarians also had lower levels of BUN (3.6 ± 1.0 mmol/L vs. 4.7 ± 5.9 mmol/L), SCr (67.8 ± 10.0 μmol/L vs. 69.5 ± 12.1 μmol/L) and UA (254.6 ± 62.9 μmol/L vs. 272.5 ± 64.3 μmol/L), which are the final metabolites and the representative parameters of renal excretion and filtration. Among vegetarians, the renal function parameters showed no differences between the vegan and lacto-ovo vegetarian groups.

As shown in Table 3, we designed five multiple-linear regression models to explore associations between vegetarian dietary patterns and renal function parameters. In a multiple-linear regression analysis using an unadjusted model, a vegetarian diet was associated with lower BUN [β = − 0.71, 95% CI: (− 0.88, − 0.53)], lower SCr [β = − 1.91, 95% CI: (− 3.72, − 0.10)], lower UA [β = − 18.41, 95% CI: (− 29.11, − 7.70)] and a higher eGFR [β = 3.06, 95% CI: (0.26, 5.85)]. After adjusting for sex, age, BMI and skeletal muscle mass, a vegetarian diet was associated with lower BUN [β = − 0.68, 95% CI: (− 0.85, − 0.51)], lower SCr [β = − 2.04, 95% CI: (− 3.51, − 0.57)], lower UA [β = − 10.17, 95% CI: (− 19.28, − 1.05)] and a higher eGFR [β = 3.59, 95% CI: (0.92, 6.28)]. In model 3, physical activity, alcohol consumption and smoking status were also controlled in addition to the variables included in model 2. After adjusting for LDL and HDL, systolic blood pressure and fasting blood glucose in model 4, a vegetarian diet was negatively associated with BUN [β = − 0.63, 95% CI: (− 0.88, − 0.38)], SCr [β = − 2.04, 95% CI: (− 4.10, 0.02)], and UA [β = − 15.15, 95% CI: (− 27.81, − 2.50)] and positively associated with the eGFR [β = 4.04, 95% CI: (0.30, 7.78)]. After adjusting for vegetarian diet duration, a vegetarian diet remained significantly associated with lower BUN, SCr, and UA levels and a higher eGFR. In a multiple-linear regression analysis, no associations were found between vegetarian diet duration and renal function parameters. Regarding different types of vegetarian diets, both lacto-ovo vegetarian diets and vegan diets were associated with lower SCr, BUN, and UA levels and significantly positively associated with higher eGFRs after adjusting for different confounders.

**Table 3:** Multiple-linear regression for the associations between vegetarian dietary patterns and renal function parameters^a^

	Omnivores	Vegetarians		
		Total vegetarians^b^	Lacto-ovo vegetarians	Vegans
		β(95% CIs)	β(95% CIs)	β(95% CIs)
BUN				
Model 1	0 (Ref)	−0.71 (− 0.88, − 0.53)	−0.76 (− 0.95, − 0.57)	−0.56 (− 0.84, − 0.27)
Model 2		− 0.68 (− 0.85, − 0.51)	− 0.71 (− 0.90, − 0.52)	− 0.58 (− 0.87, − 0.30)
Model 3		−0.72 (− 0.96, − 0.49)	−0.71 (− 0.96, − 0.46)	−0.74 (−1.13, − 0.36)
Model 4		− 0.63 (− 0.88, − 0.38)	−0.59 (− 0.86, − 0.32)	−0.64 (− 1.05, − 0.22)
Model 5		− 0.66 (− 0.95, − 0.38)	−0.66 (− 0.98, − 0.35)	−0.52 (− 1.04, 0.00)
SCr				
Model 1	0 (Ref)	−1.91 (− 3.72, − 0.10)	− 2.08 (− 4.03, − 0.12)	−1.43 (− 4.43, 1.57)
Model 2		− 2.04 (− 3.51, − 0.57)	−1.61 (− 3.12, − 0.10)	− 3.70 (− 6.08, − 1.32)
Model 3		−2.95 (− 4.85, − 1.05)	−2.46 (− 4.38, − 0.55)	− 5.41 (− 8.70, − 2.11)
Model 4		−2.04 (−4.10, 0.02)	− 1.58 (− 3.65, 0.48)	− 3.17 (− 6.24, − 0.09)
Model 5		−2.47 (− 4.83, − 0.11)	−2.41 (− 4.81, − 0.01)	−3.67 (− 8.17, − 0.86)
UA				
Model 1	0 (Ref)	− 18.41 (− 29.11, − 7.70)	− 21.72 (− 33.19, − 10.25)	−9.00 (− 26.01, − 8.03)
Model 2		− 10.17 (− 19.28, − 1.05)	−11.87 (− 21.54, − 2.19)	−5.69 (− 20.30, 8.95)
Model 3		−17.21 (− 28.76, − 5.65)	−16.47 (− 28.77, − 4.17)	−19.30 (− 37.77, − 0.82)
Model 4		− 15.15 (− 27.81, − 2.50)	− 16.29 (− 29.61, − 2.97)	−14.88 (− 35.18, 5.42)
Model 5		−18.00 (− 32.52, −3.48)	−18.73 (− 34.25, − 3.21)	− 19.96 (− 45.61, 5.68)
eGFR				
Model 1	0 (Ref)	3.06 (0.26, 5.85)	2.65 (−0.26, 5.56)	4.20 (−0.37, 8.78)
Model 2		3.59 (0.92, 6.28)	2.66 (−0.08, 5.39)	7.42 (3.02, 11.81)
Model 3		5.94 (2.48, 9.41)	4.51 (1.15, 7.87)	12.29 (6.25, 18.33)
Model 4		4.04 (0.30, 7.78)	2.69 (−0.92, 6.30)	8.31 (2.38, 13.85)
Model 5		4.55 (0.25, 8.84)	4.17 (−0.03, 8.37)	7.70 (0.53, 15.94)

^a^Model 1: Unadjusted regression; Model 2: Adjusted for sex, age, BMI and skeletal muscle mass based on Model 1; Model 3: Adjusted for physical activity, alcohol consumption and smoking status based on Model 2; Model 4: Adjusted for LDL, HDL, systolic pressure and fasting blood glucose in addition to the adjustments in Model 3; Model 5: Adjusted for vegetarian diet duration on the basis of Model 4

^b^Total vegetarians: lacto-ovo vegetarians and vegans

We designed a multiple-linear regression model to explore associations between dietary intake composition and renal function parameters. When exploring the contribution of dietary composition to renal function parameters, multiple-linear regression results showed that dietary fiber was significantly negatively associated with BUN [β = − 0.02, 95% CI: (− 0.03, 0.00)], SCr [β = − 0.12, 95% CI: (− 0.23, 0.02)], and UA [β = − 0.70, 95% CI: (− 1.34, − 0.06)] and positively associated with the eGFR [β = 0.22, 95% CI: (0.06, 0.41)]. Energy intake, the fat energy supply ratio, the protein energy supply ratio, and protein intake/weight were positively associated with BUN, and the carbohydrate energy supply ratio was negatively associated with BUN, while no convincing association was found between these factors and SCr, UA and the eGFR. To further explore the contributions of different sources of protein to renal function, we divided dietary protein into plant-sourced protein and animal-sourced protein, and the associations between different sources of protein and renal function have been discussed separately. However, we did not detect any associations between plant-sourced protein vs. animal-sourced protein and renal function parameters.

## Discussion

We found that among healthy adults, vegetarians, including lacto-ovo vegetarians and vegans, have better renal function than omnivores. Moreover, higher dietary fiber intake is the variable mainly associated with better renal function. This is the first study indicating that healthy adult vegetarians have better renal function parameters apart from the influence of blood pressure, fasting blood glucose and blood lipid levels than omnivores.

Hyperlipemia, hypertension and diabetes are well recognized as factors that influence renal function and renal diseases [19–21]. Many studies have demonstrated that compared with omnivores, vegetarians have significantly lower blood pressure, cholesterol levels, and glucose levels [2, 3]. We observed lower systolic pressure, lower fasting blood glucose and better blood lipid profile levels in vegetarians, which may be an important reason for the better renal function of vegetarians. However, after adjusting for LDL and HDL, systolic pressure and fasting blood glucose, the vegetarian diet remained significantly associated with a higher eGFR, suggesting that the vegetarian diet may have a direct influence on renal function protection.

Previous research has reported that 60–80% caloric restriction (20–40% fewer calories than an ad libitum-fed group) may yield obvious effects, including a higher eGFR [22, 23]. However, we did not detect an association between lower energy intake and a higher eGFR. Although the vegetarians had lower energy intake in our study, the degree and duration of caloric limitation may not meet the effective standard to obtain optimal benefits. We did not find a convincing association between physical activity and the eGFR. Whether physical inactivity is associated with reduced kidney function remains controversial according to previous studies [24, 25]. The association between physical activity and renal function remains to be further explored.

According to the results of the multiple-linear regression in our study, the higher dietary fiber consumption of vegetarians may contribute to better renal function. CKD is often accompanied by a chronic inflammatory state characterized by elevated serum C-reactive protein (CRP), IL-6 and TNF-alpha levels [26]. High fiber intake has been proven to reduce oxidative stress status by affecting bacterial fermentation of proteins in the colon [27, 28]. Some small-scale studies have reported that increasing fiber intake in CKD patients may reduce serum creatinine levels and improve the eGFR [29, 30]. A study among 1110 community-dwelling male participants aged 70–71 years from Sweden demonstrated that high dietary fiber intake was associated with better kidney function and lower inflammation [31].

We did not find a relationship between protein intake and the eGFR. Previous studies have confirmed the effectiveness of low total protein intake in the prevention and treatment of renal dysfunction [15, 32, 33]. However, in the context of a vegetarian diet, whether lower protein intake is associated with better renal function is difficult to assess due to changes in both the amount and source of dietary protein. On the one hand, some studies have suggested that a low-protein vegan-vegetarian diet is a suitable option in the management of CKD patients [6, 34, 35]. On the other hand, because of the lower bioavailability of vegetable proteins, a vegetarian diet may lead to a decrease in the eGFR and increase the risk of protein energy malnutrition [36, 37]. Furthermore, historical studies have shown that a greater propensity for animal-sourced protein, particularly red meat, is associated with a lower eGFR and/or kidney injury [38, 39]. However, no convincing association was found between different sources of protein and renal function parameters in our study. One possible explanation is that we failed to investigate the effects of various sources of protein, such as red and processed meat, nuts and legumes, which have been reported to be inversely or positively related to the risk of incident CKD. Our rough classification of protein sources may confound the effects. In our study, all the participants had normal protein intake meeting the recommended dietary intake standards of China regardless of dietary habits. Healthy adults have strong buffering and compensatory capabilities, which may also result in inconspicuous changes in renal metabolites and function caused by acceptable differences in protein consumption.

A few studies have shown that plant phytochemicals, such as green tea polyphenols, soy isoflavones, allicin, bitter melon extract, and platonic acid, have a strong effect on oxidative stress and help protect metabolic stability and renal excretion and filtration [40–42]. According to our previous study among vegetarians in Shanghai, a vegetarian diet was characterized by adequate consumption of whole grains, tubers, vegetables, fruits, legumes and nuts, which are rich in phytochemicals and antioxidants [43]. Vegetarian diets may contain more phytochemicals and less saturated fat and cholesterol due to the plant-based nature of the diet, resulting in a better metabolic status and milder metabolic and renal filtration burdens in vegetarians [1].

We did not detect an association between vegetarian diet duration and renal function. Previous studies have rarely addressed the role of vegetarian diet duration. According to a 24-year follow-up of 14,686 middle-aged adults, greater adherence to plant-based and vegetarian diets was associated with a slower annual eGFR decline [44], which probably indicates that maintaining a vegetarian diet can have lasting beneficial effects on renal health; thus, we suspect that a longer vegetarian diet duration may be beneficial for the maintenance and stability of kidney function. However, a study in Taiwan found no association between vegetarian diet duration and renal function, which is consistent with our results [7]. Some previous studies have confirmed that dietary fiber can improve renal function in the short term [45]. One possible explanation is that the kidney benefits of gut microbiome changes associated with dietary fiber occur in a short time rather than requiring many years of sustained fiber consumption to materialize. However, the gut microbiome is constantly changing, and long-term adherence to a vegetarian diet is required to maintain the stability of the gut microbiome. Research on the beneficial effects of dietary fiber in the CKD population in the long term is lacking. Given the limited data available, more evidence should be collected to determine the influence of vegetarian diet duration on renal function outcomes.

Some limitations of this study should be mentioned. First, the small vegetarian population in China introduced practical limitations preventing random sampling, and the relatively small sample size of vegan subjects warrants additional studies in the future. Second, due to the cross-sectional design, we could not determine the causal relationship between vegetarian diets and renal function. Third, dietary patterns may change over time, and this analysis relied on a single measurement of diet at baseline; thus, some dietary variations may not have been analyzed. In addition, the potential for uncontrolled confounding factors, such as genetic factors and unobserved lifestyle choices, remains. Because the participants in our study were young and healthy without kidney diseases, differences in kidney injury between vegetarians and nonvegetarians could not be detected. The association between dietary patterns and kidney injury requires further research, and our findings should be interpreted carefully with respect to the clinical treatment of CKD. These may be shortcomings of our study, and we will conduct additional studies in the future.

## Conclusions

In conclusion, a vegetarian diet has potential benefits for renal function that are not completely explained by the influence of glycolipid metabolism and blood pressure status. A higher intake of dietary fiber may lead to better renal function. This information may be important to advise the public about the prevention of kidney disease.

## Supplementary information

**Additional file 1.**

**Additional file 2.**

## Data Availability

The datasets used and analyzed during the current study available from the corresponding author on reasonable request.

## Abbreviations

BUN: Blood urea nitrogen
SCr: Serum creatinine
UA: Blood uric acid
eGFR: Estimated glomerular filtration rate
CKD: Chronic kidney disease
BMI: Body mass index
TC: Total cholesterol
TGs: Total triglycerides
LDL: Low-density lipoprotein cholesterol
HDL: High-density lipoprotein cholesterol
Ref: Reference
CI: Confidence interval
BMI: Body mass index

## References

[1] Rizzo NS, Jaceldo-Siegl K, Sabate J, Fraser GE (2013). Nutrient profiles of vegetarian and nonvegetarian dietary patterns - journal of the academy of nutrition and dietetics. J Acad Nutr Diet.

[2] Kahleova H, Levin S, Barnard ND. Vegetarian dietary patterns and cardiovascular disease. Prog Cardiovasc Dis. 2018:S0033062018300872. 10.1016/j.pcad.2018.05.002 29800598

[3] Davey GK, Spencer EA, Appleby PN, Allen NE, Knox KH, Key TJ (2003). EPIC-Oxford: lifestyle characteristics and nutrient intakes in a cohort of 33 883 meat-eaters and 31 546 non meat-eaters in the UK. Public Health Nutr.

[4] Sabaté J, Wien M (2015). A perspective on vegetarian dietary patterns and risk of metabolic syndrome. Br J Nutr.

[5] Giorgina Barbara P, Federica Neve V, Filomena L (2015). Low-protein diets in CKD: how can we achieve them? A narrative, pragmatic review. Clin Kidney J.

[6] Attini R, Leone F, Parisi S (2016). Vegan-vegetarian low-protein supplemented diets in pregnant CKD patients: fifteen years of experience. BMC Nephrol.

[7] Liu HW, Tsai WH, Liu JS, Kuo KL. Association of Vegetarian Diet with Chronic Kidney Disease. Nutrients. 2019;11. 10.3390/nu11020279 PMC6412429 30691237

[8] Asghari G, Momenan M, Yuzbashian E, Mirmiran P, Azizi F. Dietary pattern and incidence of chronic kidney disease among adults: a population-based study. Nutr Metab. 2018;15. 10.1186/s12986-018-0322-7 PMC6296119 30564279

[9] Chang CY, Chang HR, Lin HC, Chang HH (2018). Comparison of renal function and other predictors in lacto–Ovo vegetarians and omnivores with chronic kidney disease. J Am Coll Nutr.

[10] Turney BW, Appleby PN, Reynard JM, Noble JG, Key TJ, Allen NE (2014). Diet and risk of kidney stones in the Oxford cohort of the European prospective investigation into Cancer and nutrition (EPIC). Eur J Epidemiol.

[11] Orlich MJ, Singh PN (2013). Sabatã© J, et al. vegetarian dietary patterns and mortality in Adventist health study 2. JAMA Intern Med.

[12] Key TJ, Appleby PN, Crowe FL, Bradbury KE, Schmidt JA, Travis RC (2014). Cancer in British vegetarians: updated analyses of 4998 incident cancers in a cohort of 32,491 meat eaters, 8612 fish eaters, 18,298 vegetarians, and 2246 vegans. Am J Clin Nutr.

[13] Hwang SJ, Lin MY, Chen HC (2012). Prevalence of chronic kidney disease in China. Lancet..

[14] Li Y, Shi H, Wang WM (2016). Prevalence, awareness, and treatment of anemia in Chinese patients with nondialysis chronic kidney disease: first multicenter, cross-sectional study. Medicine..

[15] Andrassy KM (2013). Comments on ‘KDIGO 2012 clinical practice guideline for the evaluation and management of chronic kidney disease’. Kidney Int.

[16] Mula-Abed WAS, Ai Rasadi K, Ai-Riyami D (2012). Estimated glomerular filtration rate (eGFR): a serum Creatinine-based test for the detection of chronic kidney disease and its impact on clinical practice. Oman Med J.

[17] Issued by the Ministry of health of the people's Republic of China (2013). Sanitary industry standards of the People's Republic of China [M]. Standards Press of China.

[18] Yan-Ping L, Dong W, Yu-Na H (2007). Comparative study on the results of energy and nutrients intakes investigated by different evaluation methods[J]. Chin J Prev Control Chronic Non Communicable Dis.

[19] Manjula K, Lo JC, Chertow GM (2005). Metabolic syndrome and the risk for chronic kidney disease among nondiabetic adults. J Am Soc Nephrol.

[20] Tsan Y, Chi-Hong C, Chih-Hsung H (2012). Impact of metabolic syndrome on the incidence of chronic kidney disease: a Chinese cohort study. Nephrology..

[21] Leoncini G, Viazzi F, Rosei EA (2011). Chronic kidney disease in the hypertensive patient. High Blood Press Cardiovasc Prev.

[22] Xiao-Meng X, Guang-Yan C, Ru B (2015). Beneficial Effects of Caloric Restriction on Chronic Kidney Disease in Rodent Models: A Meta-Analysis and Systematic Review[J]. PLoS One.

[23] Giordani I, Malandrucco I, Donno S (2014). Acute caloric restriction improves glomerular filtration rate in patients with morbid obesity and type 2 diabetes[J]. Diabetes Metab.

[24] Gerrie-Cor M, Gast H, et al. Physical Activity Is not Associated with Estimated Glomerular Filtration Rate among Young and Middle-Aged Adults: Results from the Population-Based Longitudinal Doetinchem Study.[J]. PLoS One. 2015. 10.1371/journal.pone.0133864 PMC4605681 26465150

[25] Bharakhada N, Yates T, Davies MJ (2012). Association of Sitting Time and Physical Activity with CKD: a cross-sectional study in family practices[J]. Am J Kidney Dis.

[26] Silverstein DM (2009). Inflammation in chronic kidney disease: role in the progression of renal and cardiovascular disease. Pediatr Nephrol.

[27] Marian GB, Ming-Chin Y (2014). The health advantage of a vegan diet: exploring the gut microbiota connection. Nutrients..

[28] Salmean YA, Segal MS, Palii SP, Dahl WJ (2015). Fiber supplementation lowers plasma p -cresol in chronic kidney disease patients. J Ren Nutr.

[29] Younes H, Demigne C, Behr S, Remesy C (1995). Resistant starch exerts a lowering effect on plasma urea by enhancing urea n transfer into the large intestine. Nutr Res.

[30] Salmean YA, Segal MS, Langkamp B (2013). Foods with added Fiber lower serum Creatinine levels in patients with chronic kidney disease - journal of renal nutrition. J Ren Nutr.

[31] Hong X, Xiaoyan H, Ulf R (2014). Dietary fiber, kidney function, inflammation, and mortality risk. Clin J Am Soc Nephrol.

[32] Metzger M, Yuan WL, Haymann JP (2018). Association of a low-Protein Diet with Slower Progression of CKD. Kidney Int Rep.

[33] Rhee CM, Ahmadi SF, Kovesdy CP, Kalantar-Zadeh K. Low-protein diet for conservative management of chronic kidney disease: a systematic review and meta-analysis of controlled trials. J Cachexia Sarcopeni. 2017. 10.1002/jcsm.12264 PMC5879959 29094800

[34] Barsotti G, Morelli E, Cupisti A, Meola M, Dani L, Giovannetti S (1996). A low-nitrogen low-phosphorus vegan diet for patients with chronic renal failure. Nephron..

[35] Attini R, Leone F, Montersino B (2017). Pregnancy, proteinuria, Plant-Based Supplemented Diets and Focal Segmental Glomerulosclerosis: A Report on Three Cases and Critical Appraisal of the Literature. Nutrients.

[36] Kontessis P, Jones S, Dodds R (1990). Renal, metabolic and hormonal responses to ingestion of animal and vegetable proteins. Kidney Int.

[37] Anderson JW, Blake JE, Turner J, Smith BM (1998). Effects of soy protein on renal function and proteinuria in patients with type 2 diabetes. Am J Clin Nutr.

[38] Haring B, Selvin E, Liang M, et al. Dietary protein sources and risk for incident chronic kidney disease: results from the atherosclerosis risk in communities (ARIC) study[J]. J Ren Nutr. 2017:S1051227616301790. 10.1053/j.jrn.2016.11.004 PMC5476496 28065493

[39] Lew QLJ, Jafar TH, Koh HWL, et al. Red meat intake and risk of ESRD[J]. J Am Soc Nephrol. 2016:28(1). 10.1681/ASN.2016030248 PMC5198288 27416946

[40] Gao M, Zhao Z, Lv P (2015). Quantitative combination of natural anti-oxidants prevents metabolic syndrome by reducing oxidative stress. Redox Biol.

[41] Negrão R, Faria A (2009). Natural Polyphenols as Anti-Oxidant, Anti-Inflammatory and Anti-Angiogenic Agents in the Metabolic Syndrome// Oxidative Stress, Inflammation and Angiogenesis in the Metabolic Syndrome.

[42] Serafini M, Peluso I. Functional Foods for Health: The Interrelated Antioxidant and Anti-Inflammatory Role of Fruits, Vegetables, Herbs, Spices and Cocoa in Humans. Curr Pharm Des. 2016;22(44). 10.2174/1381612823666161123094235 PMC5427773 27881064

[43] Mao X, Shen X, Tang W, Zhao Y, Wu F, Zhu Z (2015). Prevalence of vegetarians and vegetarian’s health dietary behavior survey in Shanghai. Wei sheng Yan Jiu. J Hygiene Res.

[44] Asghari G, Momenan M, Yuzbashian E, Mirmiran P, Azizi F. Dietary pattern and incidence of chronic kidney disease among adults: a population-based study. Nutr Metab (Lond). 2018;15:88. 10.1186/s12986-018-0322-7 PMC6296119 30564279

[45] Chiavaroli L, Mirrahimi A, Sievenpiper JL (2015). Dietary fiber effects in chronic kidney disease: a systematic review and meta-analysis of controlled feeding trials[J]. Eur J Clin Nutr.

