# Supplementary material for: Healthy adult vegetarians have better renal function than matched omnivores: a cross-sectional study in China
Source: BMC Nephrol. 2020 Jul 11;21:268. doi: 10.1186/s12882-020-01918-2 (PMC7353802; doi:10.1186/s12882-020-01918-2)
Supplement: Supplementary file 1 — Additional file 1. [file 12882_2020_1918_MOESM1_ESM.docx]

Supplementary file 1:

The detailed 24h dietary recall process:

The survey was conducted by dietitians, trained by the Department of Nutrition, School of Medicine, Shanghai Jiao Tong University. All the dietitians had to pass the evaluation of professional skills. for example, the estimation error of the size of same food should not exceed 5% for each dietitian, and the estimation error of the same food quantity among different dietitians should not over 10%.

For the one-on-one, face-to-face interview, all participants were interviewed in the Department of Clinical Nutrition, Shanghai Xinhua Hospital To help the participants recall and estimate what and how much they intake during the past 24 h, the dietitians provided several assisted technologies, such as food images, verbal descriptions and food models. In case that their previous day's diet differed significantly from their typical diet (such as eating out or not eating), the most typical day in the past 72 hours was recorded.

After the interview, every dietitian double checked the questionnaire then submit it to a chief registered dietitian who was responsible for quality control. Any error should be corrected immediately.
