# Supplementary material for: Healthy adult vegetarians have better renal function than matched omnivores: a cross-sectional study in China
Source: BMC Nephrol. 2020 Jul 11;21:268. doi: 10.1186/s12882-020-01918-2 (PMC7353802; doi:10.1186/s12882-020-01918-2)
Supplement: Supplementary file 2 — Additional file 2. [file 12882_2020_1918_MOESM2_ESM.docx]

Supplementary file 2

**24-hour-dietary recall questionnaire**

Name ____________ Sex____________ Age____________ Number____________

| Time^1^ | Recipes | Ingredients | Weight of ingredient/g | Cooking methods | Dining place | Note |
| --- | --- | --- | --- | --- | --- | --- |
|  |  |  |  |  |  |  |
|  |  |  |  |  |  |  |
|  |  |  |  |  |  |  |
|  |  |  |  |  |  |  |
|  |  |  |  |  |  |  |
|  |  |  |  |  |  |  |
|  |  |  |  |  |  |  |
|  |  |  |  |  |  |  |
|  |  |  |  |  |  |  |
|  |  |  |  |  |  |  |
|  |  |  |  |  |  |  |
|  |  |  |  |  |  |  |
|  |  |  |  |  |  |  |
|  |  |  |  |  |  |  |
|  |  |  |  |  |  |  |
|  |  |  |  |  |  |  |
|  |  |  |  |  |  |  |
|  |  |  |  |  |  |  |
|  |  |  |  |  |  |  |
|  |  |  |  |  |  |  |
|  |  |  |  |  |  |  |
|  |  |  |  |  |  |  |
|  |  |  |  |  |  |  |
|  |  |  |  |  |  |  |

^1^ Time：breakfast、lunch、dinner、snacks

^2^ Dining place: home, cafeteria, restaurant, other

Interviewer：___________ Time of interview：___________
